#  Dose-Dependent Increases in Whole-Body Net Protein Balance and Dietary Protein-Derived Amino Acid Incorporation into Myofibrillar Protein During Recovery from Resistance Exercise in Older Men

**DOI:** 10.1093/jn/nxy263

**Published:** 2019-02-04

**Authors:** Andrew M Holwerda, Kevin J M Paulussen, Maarten Overkamp, Joy P B Goessens, Irene Fleur Kramer, Will K W H Wodzig, Lex B Verdijk, Luc J C van Loon

**Affiliations:** 1 NUTRIM School of Nutrition and Translational Research in Metabolism; 2 Central Diagnostic Laboratory, Maastricht University Medical Center+, The Netherlands; 3 Top Institute Food and Nutrition (TIFN), Wageningen, The Netherlands

**Keywords:** muscle protein synthesis, sarcopenia, dietary protein, exercise, dose response

## Abstract

**Background:**

Age-related decline in skeletal muscle mass is at least partly attributed to anabolic resistance to food intake. Resistance exercise sensitizes skeletal muscle tissue to the anabolic properties of amino acids.

**Objective:**

The present study assessed protein digestion and amino acid absorption kinetics, whole-body protein balance, and the myofibrillar protein synthetic response to ingestion of different amounts of protein during recovery from resistance exercise in older men.

**Methods:**

Forty-eight healthy older men [mean ± SEM age: 66 ± 1 y; body mass index (kg/m^2^): 25.4 ± 0.3] were randomly assigned to ingest 0, 15, 30, or 45 g milk protein concentrate after a single bout of resistance exercise consisting of 4 sets of 10 repetitions of leg press and leg extension and 2 sets of 10 repetitions of lateral pulldown and chest press performed at 75–80% 1-repetition maximum. Postprandial protein digestion and amino acid absorption kinetics, whole-body protein metabolism, and myofibrillar protein synthesis rates were assessed using primed, continuous infusions of l-[*ring*-^2^H_5_]-phenylalanine, l-[*ring*-^2^H_2_]-tyrosine, and l-[1-^13^C]-leucine combined with ingestion of intrinsically l-[1-^13^C]-phenylalanine and l-[1-^13^C]-leucine labeled protein.

**Results:**

Whole-body net protein balance showed a dose-dependent increase after ingestion of 0, 15, 30, or 45 g of protein (0.015 ± 0.002, 0.108 ± 0.004, 0.162 ± 0.008, and 0.215 ± 0.009 μmol Phe · kg^−1^ · min^−1^, respectively; *P* < 0.001). Myofibrillar protein synthesis rates were higher after ingesting 30 (0.0951% ± 0.0062%/h, *P* = 0.07) or 45 g of protein (0.0970% ± 0.0062%/h, *P* < 0.05) than after 0 g (0.0746% ± 0.0051%/h). Incorporation of dietary protein–derived amino acids (l-[1-^13^C]-phenylalanine) into de novo myofibrillar protein showed a dose-dependent increase after ingestion of 15, 30, or 45 g protein (0.0171 ± 0.0017, 0.0296 ± 0.0030, and 0.0397 ± 0.0026 mole percentage excess, respectively; *P* < 0.05).

**Conclusions:**

Dietary protein ingested during recovery from resistance exercise is rapidly digested and absorbed. Whole-body net protein balance and dietary protein-derived amino acid incorporation into myofibrillar protein show dose-dependent increases. Ingestion of ≥30 g protein increases postexercise myofibrillar protein synthesis rates in older men. This trial was registered at Nederlands Trial Register as NTR4492.

## Introduction

Age-related decline in skeletal muscle mass and strength, termed sarcopenia, is accompanied by impairments in functional capacity and an increased risk of developing chronic metabolic diseases (1, 2). Whereas basal muscle protein synthesis and breakdown rates appear to be unaffected by age (3, 4), the muscle protein synthetic response to the main anabolic stimuli, food intake and physical activity, appears to be blunted in older individuals (5–7). This anabolic resistance is now considered to be a key factor contributing to progressive loss of skeletal muscle mass throughout our lifespan.

A single bout of resistance exercise strongly increases muscle protein synthesis rates (8–11) and, as such, represents an effective strategy to counteract anabolic resistance. The postexercise increase in muscle protein synthesis rates can be further augmented by ingesting protein during the recovery phase (8, 9, 12). The degree to which protein ingestion stimulates muscle protein synthesis rates depends on the availability of dietary protein-derived amino acids (13). Therefore, the amount of protein ingested after exercise may largely determine the magnitude of the postexercise muscle protein synthetic response (14). So far, no study has assessed the impact of the amount of protein ingested on postprandial availability of dietary protein-derived amino acids in the circulation after resistance exercise.

Previous work has provided evidence of a dose-response relation between the amount of protein ingested and postexercise muscle protein synthesis rates in younger athletes (15, 16). In these studies, ingestion of 20 g (∼0.25 g/kg) egg (15) or whey (16) protein was shown to induce maximal postexercise muscle protein synthesis rates in younger men. In contrast, ingestion of protein doses >20 g appears to induce further increases in muscle protein synthesis rates in older individuals (13, 17, 18). Therefore, in the present study we assessed postprandial protein digestion and amino acid absorption kinetics, whole-body protein metabolism, and the muscle protein synthetic response to the ingestion of 3 different amounts of protein during recovery from a single bout of resistance exercise in older individuals. We hypothesized that a dose-response relation exists between the amount of protein ingested and the magnitude of the postprandial increase in muscle protein synthesis rates during recovery from a single bout of resistance-type exercise in healthy, older men.

## Methods

### 

#### Subjects

**FIGURE 1:**
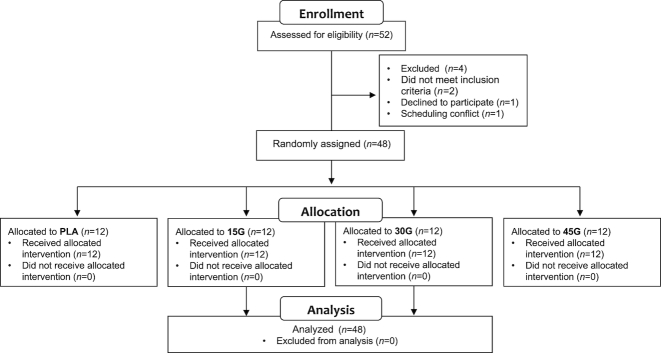
CONSORT flowchart for the human tracer infusion trial. CONSORT, Consolidated Standards of Reporting Trials; PLA, placebo; 15G, 15 g protein ingestion after resistance exercise; 30G, 30 g protein ingestion after resistance exercise; 45G, 45 g protein ingestion after resistance exercise.

A total of 48 healthy, normoglycemic, older men (age range 55–80 y, mean ± SEM 66 ± 1 y) were selected to participate in the present study. The Consolidated Standards of Reporting Trials (CONSORT) flow chart is presented in **Figure 1** and subjects’ characteristics are presented in **Table 1**. All subjects were living independently and were not participating in any structured resistance exercise training program. In a parallel design, subjects were randomly assigned to ingest either 0 g (PLA: *n* = 12), 15 g (15G: *n* = 12), 30 g (30G: *n* = 12), or 45 g (45G: *n* = 12) intrinsically l-[1-^13^C]-phenylalanine- and l-[1-^13^C]-leucine-labeled milk protein after completing a single bout of resistance exercise. All subjects were informed of the nature and possible risks of the experimental procedures before their written informed consent was obtained. The trial was conducted between July 2014 and March 2015 at Maastricht University Medical Center+, Maastricht, Netherlands. The experiments were randomized and double-blind. The study was approved by the Medical Ethical Committee of the Maastricht University Medical Center+, Netherlands, and conformed to standards for the use of human subjects in research as outlined in the most recent version of the Helsinki Declaration. The study was registered at Nederlands Trial Register as NTR4492.

**TABLE 1:** Subjects’ characteristics^1^

	PLA (*n* = 12)	15G (*n* = 12)	30G (*n* = 12)	45G (*n* = 12)	Total (*n* = 48)	*P*
Age, y	66 ± 2	69 ± 2	66 ± 1	64 ± 2	66 ± 1	0.41
Total body mass, kg	79.0 ± 3.2	78.8 ± 3.2	80.6 ± 1.5	82.7 ± 2.6	80.3 ± 1.3	0.73
Total lean mass, kg	58.1 ± 1.9	57.6 ± 2.3	61.3 ± 1.3	61.0 ± 1.8	59.5 ± 0.9	0.36
Appendicular lean mass, kg	25.9 ± 0.9	24.9 ± 1.1	27.5 ± 0.7	27.3 ± 1.0	26.4 ± 0.5	0.19
Body fat, %	23.2 ± 1.1	23.9 ± 0.9	21.7 ± 0.9	23.3 ± 1.1	23.0 ± 0.5	0.48
Height, m	1.78 ± 0.02	1.75 ± 0.02	1.80 ± 0.02	1.79 ± 0.02	1.78 ± 0.01	0.26
BMI, kg/m^2^	24.9 ± 0.6	25.8 ± 0.8	25.0 ± 0.5	25.9 ± 0.6	25.4 ± 0.3	0.62
HbA1c, %	5.5 ± 0.1	5.3 ± 0.1	5.4 ± 0.1	5.4 ± 0.1	5.4 ± 0.0	0.49
Resting plasma glucose, mmol/L	6.0 ± 0.2	5.8 ± 0.2	6.0 ± 0.2	6.1 ± 0.1	6.0 ± 0.1	0.71
Resting plasma insulin, mU/L	7.1 ± 1.3	9.3 ± 0.9	7.8 ± 2.4	7.8 ± 1.0	8.0 ± 0.7	0.77
HOMA-IR	1.9 ± 0.4	2.4 ± 0.2	2.2 ± 0.7	2.1 ± 0.2	2.1 ± 0.2	0.90
MVPA, min	139 ± 38	145 ± 31	174 ± 42	85 ± 16	136 ± 17	0.29
1-RM of leg press, kg	163 ± 8	179 ± 8	172 ± 8	193 ± 9	177 ± 4	0.09
1-RM of leg extension, kg	85 ± 4	86 ± 6	93 ± 4	96 ± 5	90 ± 2	0.35
1-RM of lat pulldown, kg	63 ± 2	60 ± 4	64 ± 2	68 ± 2	64 ± 1	0.28
1-RM of chest press, kg	55 ± 3	60 ± 6	66 ± 4	68 ± 4	62 ± 2	0.13

1 Values are means ± SEMs; *n =* 12/treatment group. Data were analyzed with a 1-factor ANOVA. No differences were detected between groups. HbA1c, glycated hemoglobin; MVPA, moderate-vigorous physical activity; PLA, placebo; resting, resting and fasted values; 1-RM, 1-repetition maximum; 15G, 15 g dietary protein; 30G, 30 g dietary protein; 45G, 45 g dietary protein.

#### Pretesting

Participants arrived at the laboratory at 0830 by car or public transport in an overnight fasted state. Upon arrival, body weight, body composition, and bone mineral content were measured with dual-energy X-ray absorptiometry (Discovery A, Hologic). Thereafter, all participants performed an oral-glucose-tolerance test. Plasma glucose and insulin concentrations were measured to determine oral glucose intolerance and the presence of type 2 diabetes mellitus according to the American Diabetes Association guidelines from 2006 (19). All subjects were screened on medical issues and excluded if any gastrointestinal, neurologic, or renal diseases were present.

Subjects were cleared to perform resistance exercise by a cardiologist who examined electrocardiograms measured at rest and during submaximal cycling (performed at 70% of age-predicted heart rate max). The subjects were then familiarized with the exercise equipment and physical activity protocol and 1-repetition maximum (1-RM) was estimated on the leg press, leg extension, lateral pulldown, and chest press exercise machines using the multiple repetitions testing procedure (20).

#### Diet and physical activity

All volunteers were instructed to refrain from any exhaustive physical activity and to keep their diet as consistent as possible for 72 h before the trial. Subjects filled in dietary records for 48 h immediately before the experimental trial. Subjects consumed 8.7 ± 0.3 MJ/d on average, consisting of 49 ± 1 energy percentage (En%) as carbohydrate, 32 ± 1 En% as fat, and 17 ± 1 En% as protein. Habitual dietary protein intake averaged 1.11 ± 0.04 g · kg^−1^· d^−1^. On the evening before the experiment, all subjects consumed a standardized meal (21.6 ± 0.4 kJ/kg, providing 55 En% as carbohydrate, 25 En% as fat, and 20 En% as protein).

#### Experimental protocol

A graphical representation of the study protocol is presented in **Supplemental Figure 1**. At 0800, participants reported to the laboratory in an overnight fasted and rested state and had Teflon catheters inserted into a vein in the antecubital space of 1 arm and a vein on the dorsal side of the opposite hand for intravenous infusion and arterialized blood sampling, respectively. At 0830 (*t* = −150 min), a background blood sample was taken before initiation of the tracer infusion protocol. The plasma and intracellular phenylalanine and leucine pools were primed with a single intravenous dose (priming dose) of l-[*ring*-^2^H_5_]-phenylalanine (3.6 µmol/kg), l-[*ring*-^2^H_2_]-tyrosine (1.10 µmol/kg), l-[1-^13^C]-leucine (7.19 µmol/kg). Once primed, a continuous stable isotope infusion was initiated (infusion rate: 0.06 µmol · kg^−1^· min^−1^l-[*ring*-^2^H_5_]-phenylalanine, 0.018 µmol · kg^−1^· min^−1^l-[*ring-*^2^H_2_]-tyrosine, 0.12 µmol · kg^−1^ · min^−1^l-[1-^13^C]-leucine; Cambridge Isotopes Laboratories). Participants rested for 1.5 h until 1000 (*t =* −60 min), at which time they completed the resistance exercise bout. At 1100 (*t =* 0 min) a blood sample was taken and a muscle biopsy was collected from a randomized leg. Subsequently, participants ingested a 500-mL beverage of PLA, 15G, 30G, or 45G with an added 1.5 mL of vanilla extract (Dr. Oetker) to improve palatability. Protein intakes represent the actual amount of protein provided in the milk protein concentrate 80 (MPC80). Blood samples (10 mL) were subsequently taken at *t =* 30, 60, 90, 120, 180, 240, 300, and 360 min after protein ingestion. A second muscle biopsy was obtained from the contralateral leg at 1700 (*t =* 360 min), signifying the end of the experimental trial. Blood and muscle samples were collected and stored as previously described (21).

#### Resistance exercise protocol

The exercise protocol consisted of 60 min of moderate-to-high intensity resistance exercise. After 10 min of cycling at 100 W with a cadence of 60–80 rotations/min, subjects performed 5 sets of 10 repetitions on the horizontal leg press machine (Technogym BV), 2 sets of 10 repetitions on the lateral pulldown machine (Technogym BV), 2 sets of 10 repetitions on the chest press machine (Technogym BV), and 5 sets of 10 repetitions on the leg extension machine (Technogym BV). The first set of each lower body exercise was performed at 50% 1-RM and sets 2–5 were performed at 75–80% 1-RM. All sets of the upper body exercises were performed at 75–80% 1-RM. Subjects were allowed to rest for 2 min between sets.

#### Preparation of tracer and production of intrinsically labeled protein

The stable isotope tracers l-[*ring-*^2^H_5_]-phenylalanine, l-[1-^13^C]-leucine, and l-[*ring-*^2^H_2_]-tyrosine were prepared as previously described (21). Intrinsically l-[1-^13^C]-phenylalanine- and l-[1-^13^C]-leucine-labeled milk protein (MPC80) was extracted from whole milk obtained during the constant infusion of l-[1-^13^C]-phenylalanine (455 µmol/min) and l-[1-^13^C]-leucine (200 µmol/min) for 96 h in a lactating dairy cow. The milk was collected, processed, and fractionated into the MPC80 as previously described (22–24). The l-[1-^13^C]-phenylalanine and l-[1-^13^C]-leucine enrichments in MPC80 averaged 38.3 mole percentage excess (MPE) and 10.8 MPE, respectively. The protein met all chemical and bacteriological specifications for human consumption.

#### Plasma and muscle analysis

Plasma glucose and insulin concentrations were analyzed using commercially available kits (GLUC3, Roche, Ref: 0,516,8791 190, and Immunologic, Roche, Ref: 12,017,547 122, respectively). Plasma amino acid concentrations and enrichments were determined by GC-MS analysis (Agilent 7890A GC/5975C; MSD). Myofibrillar protein-bound l-[*ring*-^2^H_5_]-phenylalanine enrichments were determined by GC-MS analysis, whereas the l-[1-^13^C]-phenylalanine and l-[1-^13^C]-leucine enrichments were determined by GC-C-isotope ratio mass spectrometer analysis (Trace GC Ultra, IRMS model MAT 253; Thermo Scientific). For complete details, see the Supplementary Data.

#### Calculations

Ingestion of l-[1-^13^C]-phenylalanine labeled protein, intravenous infusion of l-[*ring*-^2^H_5_]-phenylalanine, and blood sample enrichment values were used to assess whole-body phenylalanine kinetics in nonsteady state conditions. Total, exogenous, and endogenous phenylalanine rates of appearance (*R_a_*) and plasma availability of dietary protein-derived phenylalanine that appeared in the systemic circulation as a fraction of total amount of phenylalanine ingested were calculated using modified Steele's equations (25–27). For complete details, see the Supplementary Data. We simultaneously assessed the muscle protein synthetic response in steady-state conditions regardless of ingested protein dose by combining the ingestion of intrinsically l-[1-^13^C]-leucine-labeled milk protein with a primed continuous intravenous infusion of l-[1-^13^C]-leucine. The fractional synthetic rate (FSR) of myofibrillar protein was calculated by dividing the increment in muscle protein-bound l-[1-^13^C]-leucine enrichment by the precursor pool. For the latter, mean plasma l-[1-^13^C]-leucine, α-[1-^13^C]-ketoisocaproate (KIC), or muscle free l-[1-^13^C]-leucine enrichments can be applied. Consequently, myofibrillar FSR is calculated as follows (27):

(1) \begin{equation*}
{\rm FSR}\ \left( {\% /{{\rm{h}}}} \right) = \left( {\frac{{{E_{m2}} - \ {E_{m1}}}}{{{E_{\rm precursor}} \times \ t}}} \right) \times 100
\end{equation*}

*E_m_*_2_*− E_m_*_1_ represents the change in muscle protein-bound l-[1-^13^C]-leucine enrichment. *E*_precursor_ represents the average plasma l-[1-^13^C]-leucine, α-[1-^13^C]-KIC, or muscle free L-[1-^13^C]-leucine enrichment during the tracer incorporation period. *t* indicates the time interval (hours) between biopsies.

#### Statistics

A sample size of 48 (12 subjects/group) was calculated a priori based on an effect size of 0.5 for FSR between groups (9, 13), a significance level of 0.05, and a power of 0.8. All data are expressed as means ± SEMs. Baseline characteristics between groups were compared using 1-factor ANOVA. A 2-factor repeated measures ANOVA (time × treatment) with time as within-subjects factor and treatment group as between-subjects factor, was performed for analysis of plasma glucose and insulin concentrations, plasma amino acid concentrations, plasma tracer enrichments, and whole-body protein kinetics. The analysis was carried out for the period starting at the time of protein administration, between *t =* 0 and 360 min. Only on identification of a significant time × treatment interaction were separate analyses performed using 1-factor ANOVA with Tukey post hoc testing to identify time points at which the treatments differed, and 1-factor repeated measures ANOVA to identify changes over time within the separate groups. Nontime-dependent variables (i.e., whole-body protein metabolism, FSR values, l-[1-^13^C]-phenylalanine myofibrillar enrichments, AUC, peak values) were compared between treatment groups using a 1-factor ANOVA. If a significant group effect was detected using the 1-factor ANOVA, Tukey post hoc testing was used to identify how the groups significantly differed from one another. Statistical significance was set at *P* < 0.05. All calculations were performed using SPSS 21.0 (IBM).

## Results

### 

#### Plasma concentrations

Plasma glucose (**Supplemental Figure 2**A) concentration decreased after beverage ingestion (*P* < 0.001), but did not differ between groups. Plasma insulin concentration (Supplemental Figure 2B) increased rapidly after protein ingestion (*P* < 0.001), and was significantly greater at *t* = 30 in the 45G group compared with the PLA group (*P* = 0.002).

**FIGURE 2:**
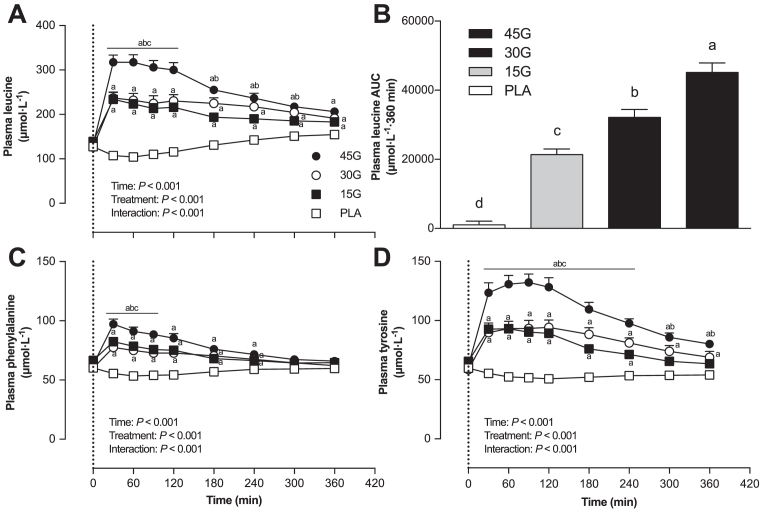
Plasma leucine (A), phenylalanine (C), and tyrosine (D) concentrations after ingestion of PLA, 15G, 30G, or 45G after resistance exercise in older men. The dotted line represents the ingestion of the beverage. Values represent means ± SEMs, *n* = 12. Data were analyzed with repeated measures (time × treatment group) ANOVA and separate analyses when a significant interaction was detected (see Methods section). For panels A, C, and D: ^a^Different from PLA at that time (*P* < 0.05); ^b^Different from 15G at that time (*P* < 0.05); ^c^Different from 30G at that time (*P* < 0.05). Plasma leucine AUC (B) over 360 min (μmol · 360 min/L) were analyzed with use of a 1-factor ANOVA. Tukey post hoc testing was used to detect differences between groups. For panel B: labeled means without a common letter differ (*P* < 0.05). PLA, placebo; 15G, 15 g protein ingestion after resistance exercise; 30G, 30 g protein ingestion after resistance exercise; 45G, 45 g protein ingestion after resistance exercise.

Plasma leucine concentration (**Figure 2**A) increased rapidly after protein ingestion (*P* < 0.001), and was greater in the 45G group compared with the 30G and 15G groups (*P* < 0.001). AUC analyses (Figure 2B) revealed a dose-dependent increase in plasma leucine availability over the 6-h postprandial period based on the amount of protein ingested (*P* < 0.001). Plasma phenylalanine concentration (Figure 2C) increased rapidly after protein ingestion (*P* < 0.001), and was greater in the 45G group compared with the 30G and 15G groups at *t* = 30, 60, and 90 min (all *P* < 0.05). Plasma tyrosine concentration (Figure 2D) increased after protein ingestion (*P* < 0.001), and was higher in the 45G group compared with the PLA group at *t =* 30, 60, 90, 120, 180, and 240 min (all *P* < 0.01).

#### Plasma amino acid enrichments

**FIGURE 3:**
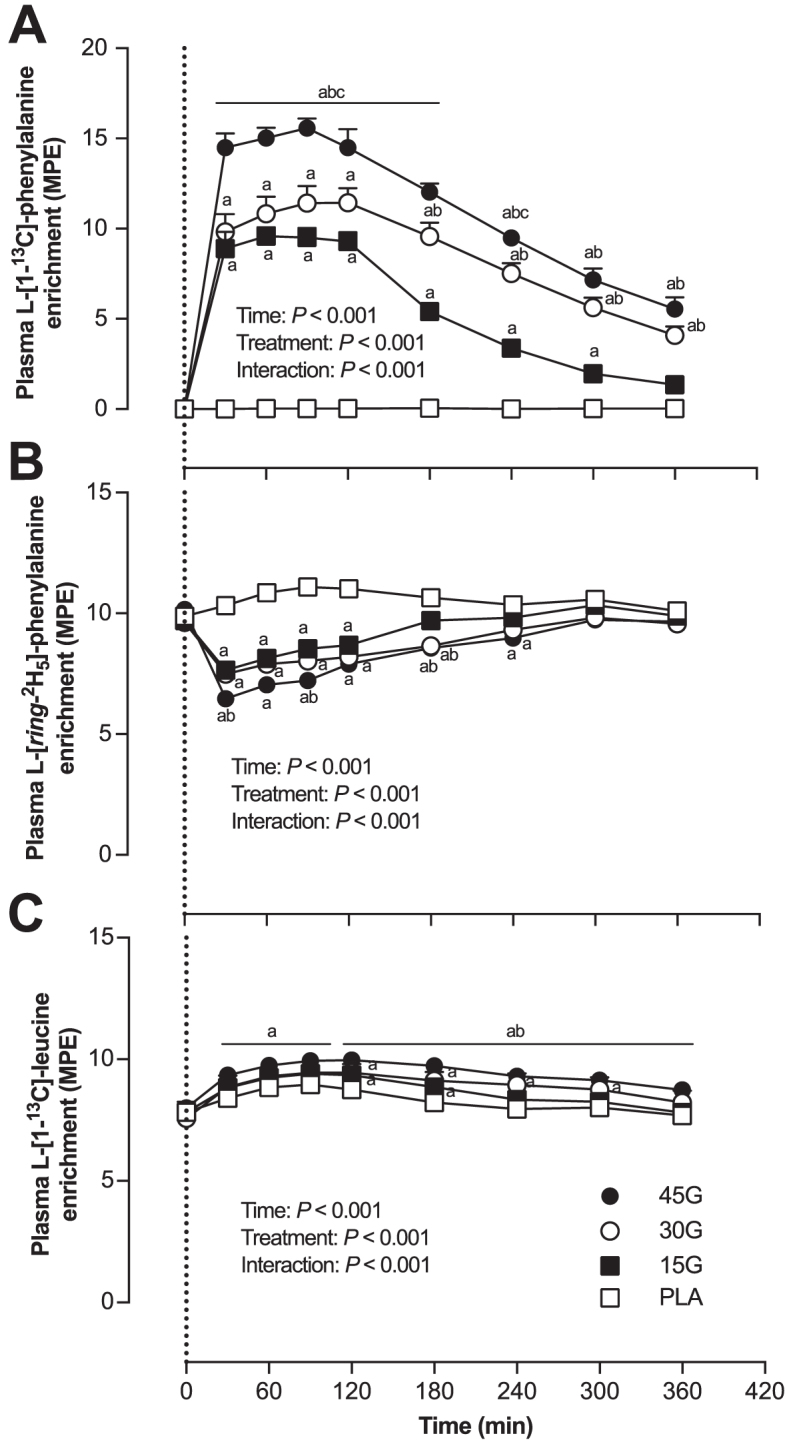
Plasma l-[1-^13^C]-phenylalanine (A), l-[*ring*-^2^H_5_]-phenylalanine (B), and l-[1-^13^C]-leucine (C) enrichments after ingestion of PLA, 15G, 30G, or 45G after resistance exercise in older men. The dotted line represents the ingestion of the beverage. Values represent means ± SEMs, *n* = 12. Data were analyzed with repeated measures (time × treatment group) ANOVA and separate analyses when a significant interaction was detected (see Methods section). ^a^Different from PLA at that time (*P* < 0.05); ^b^Different from 15G at that time (*P* < 0.05); ^c^Different from 30G at that time (*P* < 0.05). MPE, mole percentage excess; PLA, placebo; 15G, 15 g protein ingestion after resistance exercise; 30G, 30 g protein ingestion after resistance exercise; 45G, 45 g protein ingestion after resistance exercise.

Plasma enrichment from ingested (l-[1-^13^C]-phenylalanine; **Figure 3**A), infused (l-[*ring*-^2^H_5_]-phenylalanine; Figure 3B), and ingested and infused (l-[1-^13^C]-leucine; Figure 3C) amino acid tracers did not differ between treatments before protein ingestion (*t =* 0 min; *P* > 0.05 for all tracers). However, after protein ingestion, plasma l-[1-^13^C]-phenylalanine enrichment, originating from the ingested protein, increased in all protein groups, reaching peak values at *t =* 60 min in the 15G group, *t =* 120 min in the 30G group, and *t* = 90 min in the 45G group. Plasma l-[1-^13^C]-phenylalanine enrichment in the 15G group was greater than enrichment in the PLA group until *t =* 360 min. Increases in plasma l-[1-^13^C]-phenylalanine enrichments in the 30G and 45G groups were sustained above that of the PLA group for the duration of the 6-h postprandial period (*P* < 0.001 for all time points). Plasma l-[*ring*-^2^H_5_]-phenylalanine enrichment was higher in the PLA group compared with all the protein groups (*P* < 0.001), more specifically until *t* = 240 min compared with the 15G group and *t* = 300 min compared with the 30G and 45G groups. Plasma l-[1-^13^C]-leucine enrichment was lower in the PLA group compared with the 30G (*P* = 0.040) and the 45G groups (*P* < 0.001), and also was lower in the 15G group compared with the 45G group (*P* = 0.010).

#### Whole-body amino acid kinetics

**FIGURE 4:**
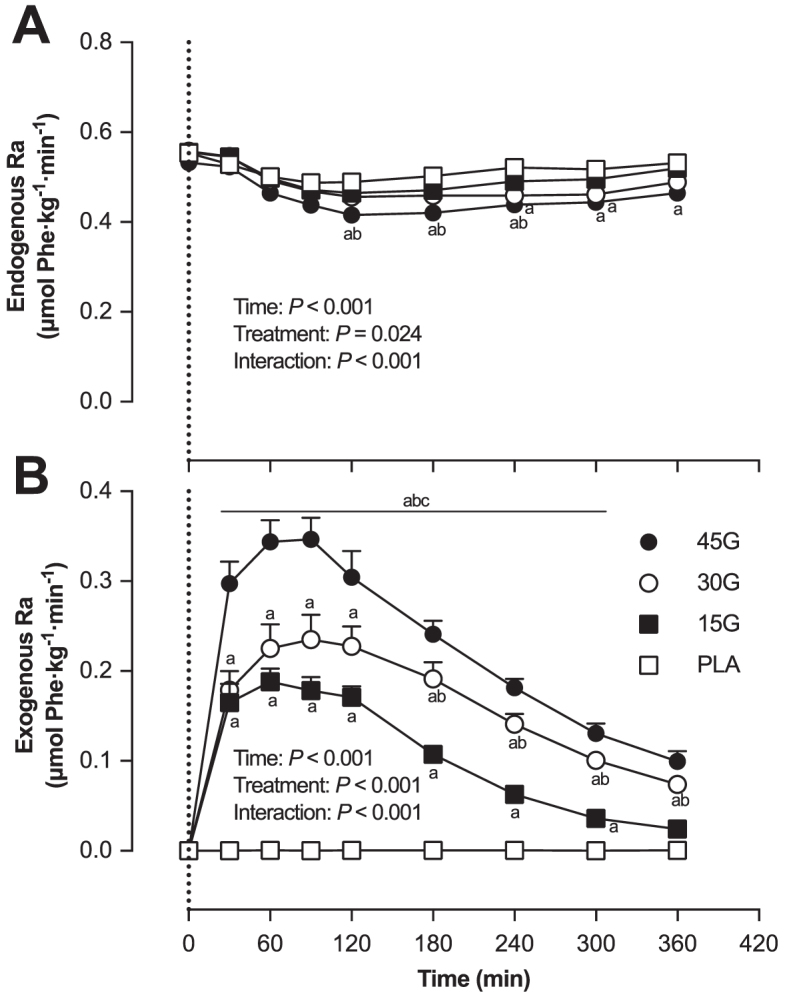
Endogenous phenylalanine rate of appearance (Ra) (A) and exogenous phenylalanine Ra (B) after ingestion of PLA, 15G, 30G, or 45G after resistance exercise in older men. The dotted line represents the ingestion of the beverage. Values represent means ± SEMs, *n* = 12. Data were analyzed with repeated measures (time x treatment group) ANOVA and separate analyses when a significant interaction was detected (see Methods section). Tukey post hoc testing was used to detect differences between groups. ^a^Different from PLA at that time (*P* < 0.05); ^b^Different from 15G at that time (*P* < 0.05); ^c^Different from 30G at that time (*P* < 0.05). PLA, placebo; Ra, total rate of appearance; 15G, 15 g protein ingestion after resistance exercise; 30G, 30 g protein ingestion after resistance exercise; 45G, 45 g protein ingestion after resistance exercise.

Endogenous phenylalanine *R_a_* (**Figure 4**A) was significantly lower after protein intake in the 45G group compared with the PLA group (*P* = 0.018). Exogenous phenylalanine *R_a_* (Figure 4B) increased after protein intake in all protein groups (*P* < 0.001), and was higher in the 45G group compared with the 15G and 30G groups (*P* < 0.001).

**FIGURE 5:**
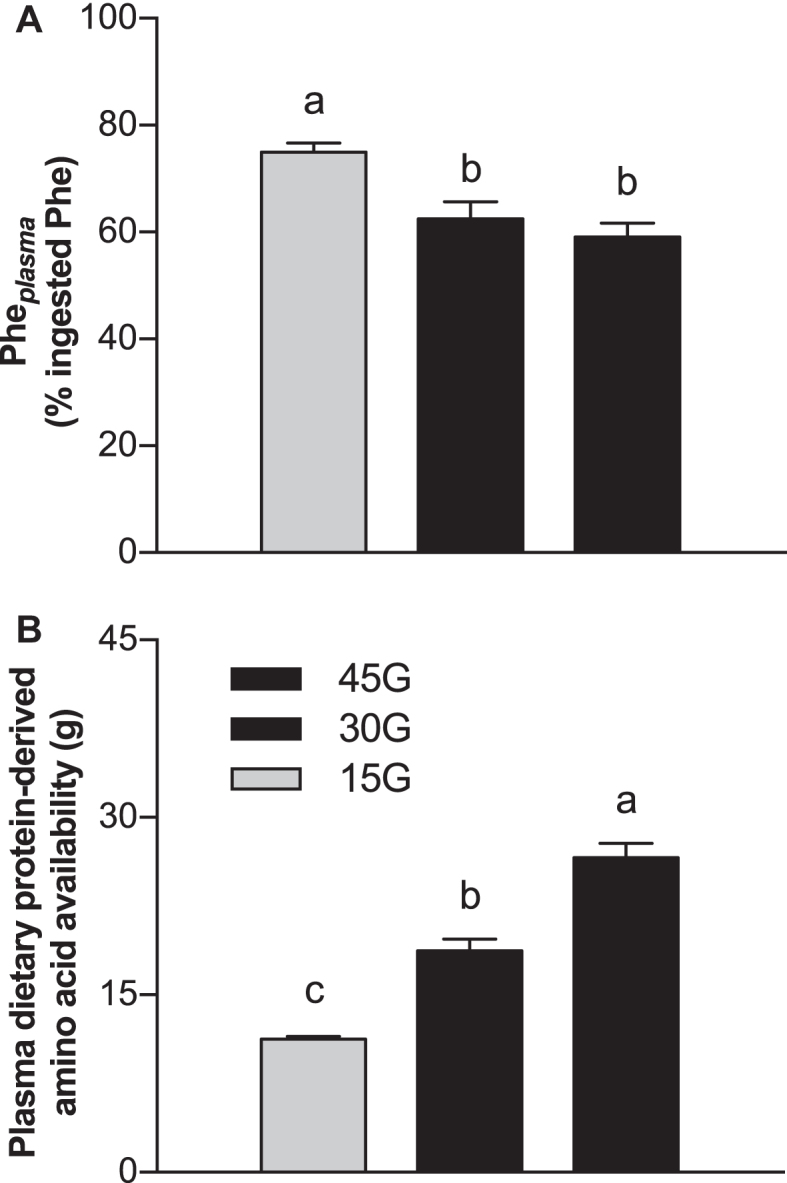
Calculated relative Phe*_plasma_* (A) of total ingested phenylalanine, and estimated absolute dietary protein–derived amino acid availability (B) after ingestion of 15G, 30G, or 45G after resistance exercise in older men. Values represent means ± SEMs, *n* = 12. Data were analyzed with 1-factor ANOVA. Tukey post hoc testing was used to detect differences between groups. Labeled means without a common letter differ (*P* < 0.05). Phe*_plasma_*, plasma phenylalanine availability; 15G, 15 g protein ingestion after resistance exercise; 30G, 30 g protein ingestion after resistance exercise; 45G, 45 g protein ingestion after resistance exercise.

Dietary protein-derived phenylalanine availability, calculated as a fraction of the total amount of ingested phenylalanine (**Figure 5**A), was higher in the 15G group compared with the 30G and 45G groups (*P* < 0.001 for both). The absolute amount of dietary protein-derived phenylalanine that appeared in the circulation during the 6-h postprandial period (Figure 5B) increased with increasing amount of ingested protein (*P* < 0.001).

**FIGURE 6:**
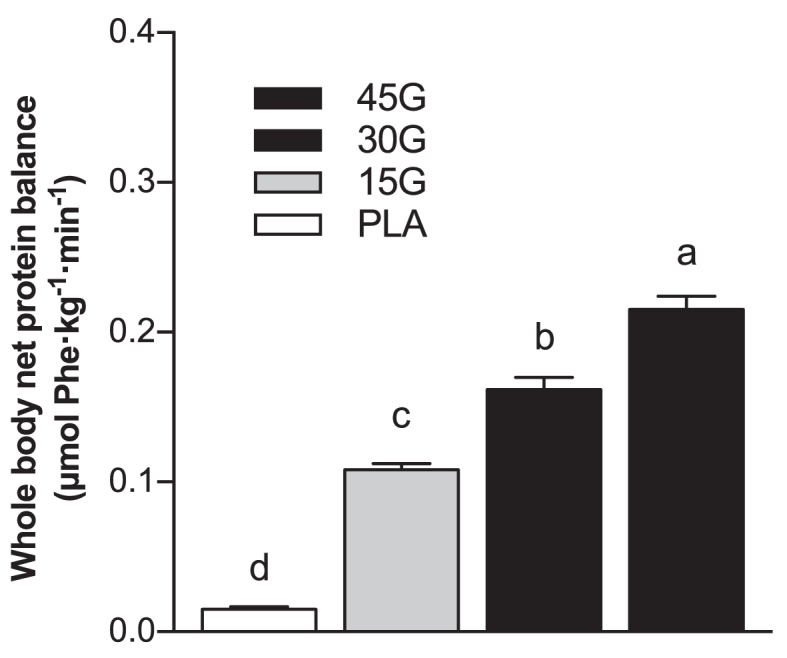
Calculated rates of whole-body net protein balance after ingestion of PLA, 15G, 30G, or 45G after resistance exercise in older men. Values represent means ± SEMs, *n* = 12. Data were analyzed with 1-factor ANOVA. Tukey post hoc testing was used to detect differences between groups. Labeled means without a common letter differ (*P* < 0.05). PLA, placebo; 15G, 15 g protein ingestion after resistance exercise; 30G, 30 g protein ingestion after resistance exercise; 45G, 45 g protein ingestion after resistance exercise.

Whole-body protein synthesis rates increased after protein ingestion (*P* < 0.01 for all groups), with greater rates in the 45G group compared with the 15G group (15G: 0.60 ± 0.01 μmol Phe · kg^−1^· min^−1^; 30G: 0.64 ± 0.01 μmol Phe · kg^−1^· min^−1^; 45G: 0.66 ± 0.02 μmol Phe · kg^−1^· min^−1^; *P* = 0.038). Whole-body protein breakdown rates were lower in the 45G group (0.45 ± 0.01 μmol Phe · kg^−1^· min^−1^) compared with the PLA group (0.51 ± 0.02 μmol Phe · kg^−1^· min^−1^, *P* = 0.004). Whole-body protein oxidation rates were greater in the 45G group (0.060 ± 0.003 μmol Phe · kg^−1^· min^−1^) compared with the PLA (0.046 ± 0.002 μmol Phe · kg^−1^· min^−1^, *P* = 0.002) and 15G groups (0.049 ± 0.013 μmol Phe · kg^−1^· min^−1^, *P* = 0.034). In all protein groups, whole-body net protein balance (**Figure 6**) was positive during the 6-h postprandial period, and greater compared with the PLA group (*P* < 0.001 for all protein groups), with a higher net balance observed in the 45G group than the 30G and 15G groups (*P* < 0.001). Whole-body net protein balance was also higher in the 30G group compared with the 15G group (*P* < 0.001).

#### Myofibrillar fractional synthesis rates and protein-bound enrichments

**FIGURE 7:**
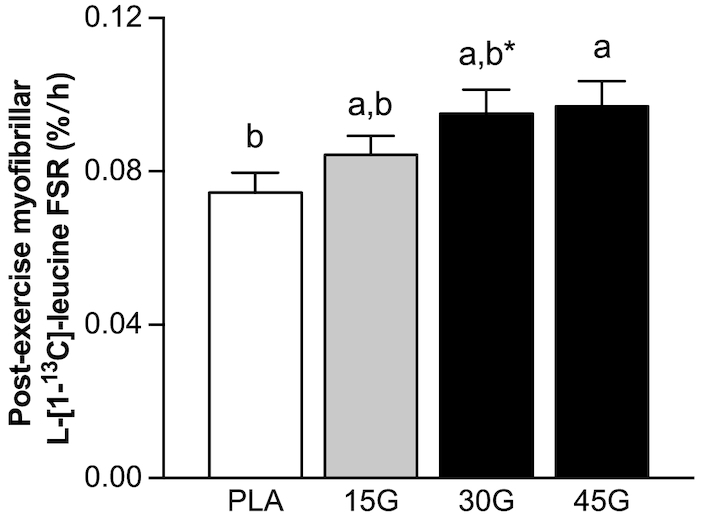
Postexercise myofibrillar protein fractional synthetic rates assessed using l-[1-^13^C]-leucine after ingestion of PLA, 15G, 30G, or 45G in older men. Values represent means ± SEMs, *n* = 12. Data were analyzed with 1-factor ANOVA. Tukey post hoc testing was used to detect differences between groups. Labeled means without a common letter differ (*P* < 0.05). *Trend for a significant difference (*P* < 0.10) from PLA. FSR, fractional synthetic rate; PLA, placebo; 15G, 15 g protein ingestion after resistance exercise; 30G, 30 g protein ingestion after resistance exercise; 45G, 45 g protein ingestion after resistance exercise.

**FIGURE 8:**
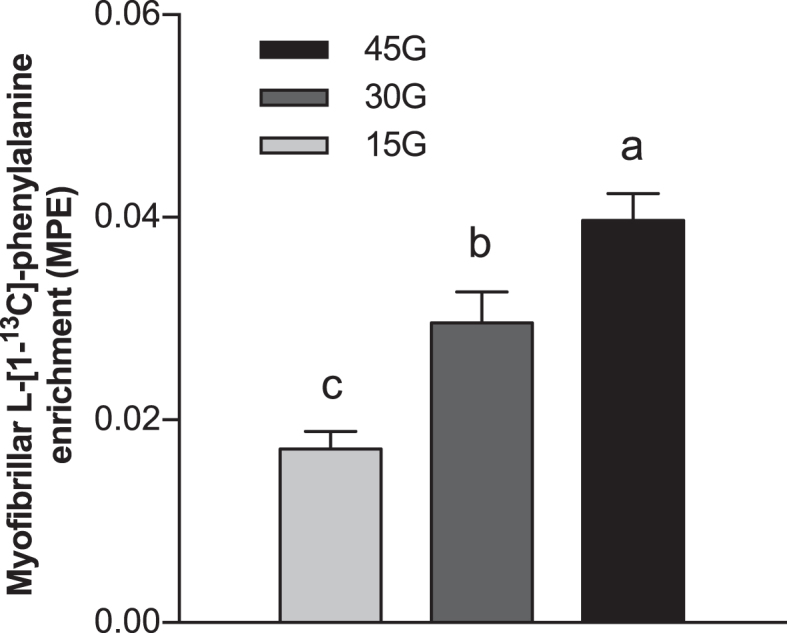
l-[1-^13^C]-phenylalanine incorporation into myofibrillar protein after ingestion of 15G, 30G, or 45G after resistance exercise in older men. Values represent means ± SEMs, *n* = 12. Data were analyzed with a 1-factor ANOVA. Tukey post hoc testing was used to detect differences between groups. Labeled means without a common letter differ (*P* < 0.05). MPE, mole percentage excess; 15G, 15 g protein ingestion after resistance exercise; 30G, 30 g protein ingestion after resistance exercise; 45G, 45 g protein ingestion after resistance exercise.

Myofibrillar l-[1-^13^C]-leucine enrichment was measured in muscle samples collected immediately before protein ingestion and after the 6-h postprandial period. The postprandial increase in myofibrillar protein-bound l-[1-^13^C]-leucine enrichment averaged: 0.0286 ± 0.0016, 0.0314 ± 0.0016, 0.0381 ± 0.0027, and 0.0414 ± 0.0025 MPE for the PLA, 15G, 30G, and 45G groups, respectively (*P* < 0.001), with higher enrichment in the 45G group than the PLA (*P* = 0.001) and 15G (*P* = 0.012) groups, and higher enrichment in the 30G group than the PLA group (*P* = 0.018). Myofibrillar protein FSR (in %/h) was calculated using the increase in myofibrillar protein-bound l-[1-^13^C]-leucine enrichment and the weighted average of plasma l-[1-^13^C]-leucine, α-[1-^13^C]-KIC, or tissue free l-[1-^13^C]-leucine enrichment during the tracer incorporation period. Myofibrillar protein FSR calculated using muscle free l-[1-^13^C]-leucine was 28% greater in the 30G group than in the PLA group (*P* = 0.069), and 30% greater in the 45G group than in the PLA group (**Figure 7**; *P* = 0.040). Myofibrillar protein FSR calculated using plasma l-[1-^13^C]-leucine averaged 0.0574% ± 0.0037%, 0.0598% ± 0.0030%, 0.0700% ± 0.0048%, and 0.0725% ± 0.0048%/h, for the PLA, 15G, 30G, and 45G groups, respectively (*P* = 0.021), with a statistically significant difference detected between the PLA and 45G groups (*P* = 0.044). Myofibrillar protein FSR calculated using plasma α-[1-^13^C]-KIC averaged 0.0655% ± 0.0039%, 0.0649% ± 0.0033%, 0.0743% ± 0.0052%, and 0.0747% ± 0.0052%/h, for the PLA, 15G, 30G, and 45G groups, respectively. l-[1-^13^C]-phenylalanine myofibrillar protein-bound enrichment (**Figure 8**) was dose-dependent, with statistically significant differences detected between groups (*P* < 0.05 for each comparison).

## Discussion

In the present study, we assessed postprandial protein digestion and amino acid absorption kinetics, whole-body protein metabolism, and the muscle protein synthetic response to the ingestion of different amounts of protein after a single bout of resistance exercise in older men. We observed that protein ingested after resistance exercise was normally digested and absorbed, with 60–75% of the ingested protein-derived phenylalanine appearing in the circulation throughout a 6-h postexercise recovery period. Ingestion of 30 or 45 g protein resulted in greater postexercise myofibrillar protein synthesis rates compared with ingestion of a placebo. Ingesting greater amounts of protein resulted in more dietary protein-derived phenylalanine being directed towards de novo muscle protein synthesis.

The ingestion of intrinsically l-[1-^13^C]-phenylalanine labeled protein combined with intravenous administration of l-[*ring*-^2^H_5_]-phenylalanine allowed us to assess dietary protein digestion and amino acid absorption in response to ingestion of different amounts of protein during recovery from exercise in older men (22, 28). Upon protein ingestion, we observed a rapid rise in circulating plasma amino acid concentrations (Figure 2) and l-[1-^13^C]-phenylalanine enrichment (Figure 3A), demonstrating rapid protein digestion and subsequent absorption of dietary protein-derived phenylalanine during postexercise recovery. Over the entire 6-h postexercise recovery period, total dietary protein-derived phenylalanine released in the circulation was 75% ± 2%, 62% ± 3%, and 59% ± 3% after ingestion of 15, 30, or 45 g protein, respectively. Previous work has suggested that more dietary-derived amino acids are retained by splanchnic tissues during first-pass when greater amounts of protein are consumed (13, 29, 30). This is the first study to quantitate the dose-response to different amounts of phenylalanine ingested as milk protein and the subsequent postprandial release of dietary protein-derived phenylalanine released in the circulation during recovery from exercise. Through the use of labeled phenylalanine, an estimated total of 3.8 ± 0.3, 11.3 ± 1.0, and 18.4 ± 1.2 g protein-derived amino acids were not released in the circulation during the 6-h postprandial period after ingestion of 15, 30, or 45 g protein, respectively. However, despite greater relative and absolute dietary protein-derived phenylalanine retention, more dietary protein-derived phenylalanine was released after ingestion of the greater amounts of protein (representing an estimated 11.2 ± 0.3, 18.7 ± 1.0, and 26.6 ± 1.2 g amino acids after ingestion of 15, 30, or 45 g protein). Consequently, a greater postprandial rise in plasma amino acid concentration occurred after ingestion of the greater amounts of protein, with the greatest peak exogenous phenylalanine *R_a_* seen at *t* = 90 min after ingestion of 45 g protein (Figure 4B). Peak plasma leucine concentration was greater after ingestion of 45 g protein (317 ± 16 μmol/L) than after ingestion of the other protein doses. Ingestion of greater protein amounts also resulted in greater peak plasma insulin concentration, with ingestion of 45 g protein resulting in the greatest peak insulin concentrations compared with placebo (Supplemental Figure 2B). Altogether, these data demonstrate that ingestion of larger amounts of protein allow greater exogenous amino acid availability, resulting in a greater postprandial rise in plasma concentrations of leucine and insulin, both important factors driving the postprandial rise in muscle protein synthesis rates.

Primed continuous infusions of l-[*ring*-^2^H_5_]-phenylalanine (and [6,6–^2^H_2_]-tyrosine) allow for the calculation of protein synthesis, breakdown, and oxidation rates on a whole-body level, providing a measure of whole-body protein balance. Protein ingestion increases whole-body net protein balance (22, 31). In the present study, protein ingestion resulted in a (more) positive whole-body net protein balance during postexercise recovery, which further increased when greater amounts of protein were ingested (Figure 6). This observation aligns with previous work assessing protein and/or nitrogen balance under resting conditions by our research group (13) as well as others (32–35), and demonstrates that whole-body net protein balance does not appear to reach an upper limit within the range of protein amounts studied (36). Ingestion of 45 g protein increased whole-body amino acid oxidation rates compared with PLA and 15 g protein. These findings are in line with previous studies conducted in both younger and older individuals (15–18) and demonstrate that more amino acids are directed towards oxidation after ingestion of larger amounts of protein. It should be noted, however, that the postprandial rise in whole-body protein synthesis rate, greater whole-body protein balance, and whole-body amino acid oxidation do not necessarily reflect the anabolic response in skeletal muscle tissue.

By combining ingestion of l-[1-^13^C]-leucine-labeled milk protein with continuous intravenous l-[1-^13^C]-leucine infusions, we were able to maintain isotopic steady state regardless of the amount of protein ingested (Figure 3C). This approach allows for proper comparison of fractional myofibrillar protein synthetic responses between treatment groups (Figure 7). Ingestion of 15 g (∼0.19 g/kg) milk protein did not significantly increase postexercise myofibrillar protein synthesis rates compared with ingestion of a non-protein placebo. Recent work has shown that healthy older individuals may not be anabolically resistant to the ingestion of a small amount (6.7 g) of free essential amino acids (representing the amount of essential amino acids in ∼20 g milk protein) under resting conditions (37). Ingestion of free essential amino acids, as opposed to intact protein, results in a greater and more rapid release of exogenous amino acids into the circulation. Therefore, ingestion of free essential amino acids may provide a more potent anabolic stimulus for skeletal muscle tissue. However, free essential amino acid ingestion is not part of a normal dietary pattern. As such, it is more relevant to compare the present study with others that have provided complete protein sources. For example, the present findings appear to be in contrast with previous work, which demonstrated that ingestion of 20 g (∼0.25 g/kg) whey protein increases postexercise muscle protein synthesis rates in healthy older men (17). The apparent discrepancy may be attributed to the smaller amount of protein ingested (15 compared with 20 g) and/or the use of a milk protein concentrate, which contains only 20% whey protein. In the present study, ingestion of 30 (∼0.37 g/kg) and 45 g (∼0.55 g/kg) protein increased myofibrillar protein synthesis rates compared with placebo ingestion. Our findings seem to be in contrast to recent studies conducted in younger men, demonstrating that postexercise muscle protein synthesis rates reach maximal values after ingestion of 20 g (∼0.25 g/kg) egg (15) or whey (38) protein, with no further increase after ingestion of 40 g protein. The apparent discrepancy between responses in young and older adults agrees with the concept of anabolic resistance, with older individuals demonstrating a more blunted muscle protein synthetic response to the ingestion of ≤20 g (∼0.25 g/kg) protein in comparison with younger controls (5, 7, 13). However, as demonstrated here and in 2 recent studies (17, 18), older individuals retain the capacity to increase myofibrillar protein synthesis rates after exercise but seem to require more dietary protein to allow a substantial increase in postexercise muscle protein synthesis rates compared with younger individuals.

In the present study, participants ingested specifically produced intrinsically l-[1-^13^C]-phenylalanine-labeled protein at a very high enrichment (38 MPE), allowing us to directly assess the metabolic fate of the dietary protein-derived phenylalanine. We demonstrate that amino acids originating from the ingested protein are used for de novo skeletal muscle protein synthesis during postexercise recovery, as illustrated by the increase in myofibrillar protein-bound l-[1-^13^C]-phenylalanine (Figure 8). Ingestion of greater amounts of protein resulted in a dose-dependent increase in dietary protein-derived l-[1-^13^C]-phenylalanine incorporation into skeletal muscle protein during postexercise recovery.

It is well established that the anabolic response to protein ingestion is blunted in both healthy (5, 7, 39) and more clinically compromised older populations, such as those living with sarcopenia (5, 40–42). This anabolic resistance can be compensated for by combining resistance exercise with protein ingestion (9, 17). Therefore, resistance exercise training and consumption of adequate protein during postexercise recovery likely represent one of the most effective strategies for counteracting the progression of sarcopenia. The amount of protein ingested after exercise is arguably the key nutritional factor dictating the magnitude of the postexercise muscle protein synthetic response (14). The present study shows that ingestion of ≥30 g (∼0.37 g/kg) protein results in a measurable rise in postexercise muscle protein synthesis rates compared with placebo, with ingestion of 45 g (∼0.55 g/kg) protein providing a greater amount of dietary protein-derived phenylalanine for de novo muscle protein synthesis. Our findings indicate that older men may need to consume well above the much-advised 20 g (∼0.25 g/kg) of a high-quality protein source (e.g., whey) to substantially increase postexercise muscle protein synthesis rates (14, 43). However, ingesting 30–45 g (∼0.37–0.55 g/kg) protein in a single mixed meal may be practically challenging for the older population (44, 45). Furthermore, the anabolic response to ingestion of a mixed meal may be modulated by co-ingestion of nondairy protein sources (46–48) and interaction of protein with other nutrients (22, 49, 50). Therefore, active older individuals may consider consuming more protein-dense foods and/or fortifying the postexercise meal with an isolated protein source to ensure protein intake beyond 30 g (∼0.37 g/kg).

In conclusion, dietary protein ingested after resistance exercise is rapidly digested and absorbed, with an estimated 60–75% of the protein-derived amino acids being released in the circulation after ingestion of 15, 30, or 45 g protein. Whole-body net protein balance and dietary protein-derived amino acid incorporation into myofibrillar protein show dose-dependent increases. Ingestion of ≥30 g (∼0.37 g/kg) protein increases postexercise myofibrillar protein synthesis rates in older men.

## Supplementary Material

nxy263_Supplement_Files

## Notes

Supported by Top Institute Food and Nutrition (TIFN), a public-private partnership on precompetitive research in food and nutrition. The researchers are responsible for the study design, data collection and analysis, decision to publish, and preparation of the manuscript. The industrial partners have contributed to the project through regular discussion.

Author disclosures: AMH, KJMP, MO, JPBG, IFK, and WKWHW, no conflicts of interest. LBV received speaker's fees from Friesland Campina and Nutricia Research. LJCvL has received research grants, consulting fees, speaking honoraria, or a combination of these, from Friesland Campina, Nutricia Research, and PepsiCo. All other authors declare no conflicts of interest.

Supplemental Figures 1 and 2 are available from the “Supplementary data” link in the online posting of the article and from the same link in the online table of contents at https://academic.oup.com/jn/.

Abbreviations used: En%, energy percentage; FSR, fractional synthetic rate; MPC80, milk protein concentrate 80; MPE, mole percentage excess; PLA, placebo ingestion after resistance exercise; R*a*, total rate of appearance; 1-RM, 1-repetition maximum; 15G, 15 g protein ingestion after resistance exercise; 30G, 30 g protein ingestion after resistance exercise; 45G, 45 g protein ingestion after resistance exercise; α-[1-^13^C]-KIC, α-[1-^13^C]-ketoisocaproate.
