# Supplementary material for: Dose-Dependent Increases in Whole-Body Net Protein Balance and Dietary Protein-Derived Amino Acid Incorporation into Myofibrillar Protein During Recovery from Resistance Exercise in Older Men
Source: J Nutr. 2019 Feb 4;149(2):221–30. doi: 10.1093/jn/nxy263 (PMC6374151; doi:10.1093/jn/nxy263)
Supplement: nxy263_Supplement_Files [file nxy263_supplement_files.zip › Sup. Figure 1 - Study scheme.pdf]

Supplementary data

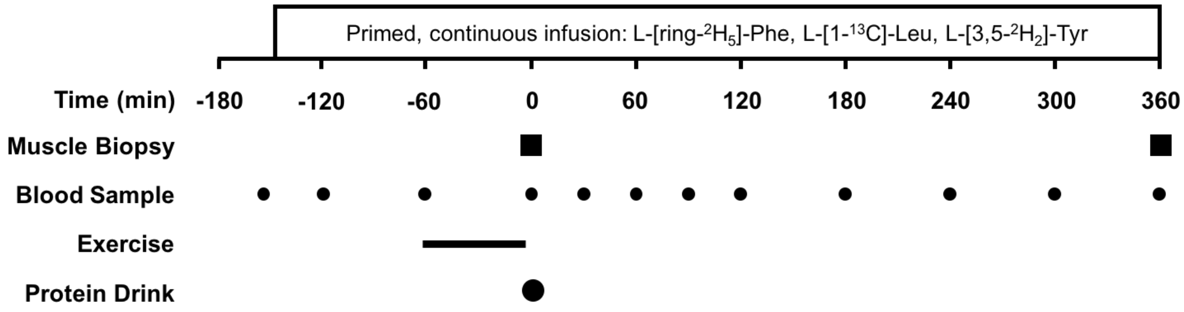

Supplemental Figure 1. Graphical representation of the experimental protocol.
