# Supplementary material for: Dose-Dependent Increases in Whole-Body Net Protein Balance and Dietary Protein-Derived Amino Acid Incorporation into Myofibrillar Protein During Recovery from Resistance Exercise in Older Men
Source: J Nutr. 2019 Feb 4;149(2):221–30. doi: 10.1093/jn/nxy263 (PMC6374151; doi:10.1093/jn/nxy263)
Supplement: nxy263_Supplement_Files [file nxy263_supplement_files.zip › Sup. Figure 2 - Glu-Ins.docx]

**Supplemental Figure 2.** Plasma glucose (A) and insulin concentrations (B) following ingestion of 0 (PLA; *n*=12), 15 (15G; *n*=12), 30 (30G; *n*=12) 45 (45G; *n*=12) g of milk protein concentrate after resistance exercise in older men. The dotted line represents the ingestion of the beverage. Values represent means+SEM. Data were analyzed with a repeated measure (time x treatment group) ANOVA and separate analyses when a significant interaction was detected (see Methods section). ^a^ Significant difference from PLA within same time point (*P*<0.05).
